# Incidence, characteristics, and consequences of fractures after acute ischemic stroke and TIA—A prospective cohort study

**DOI:** 10.1177/17474930251345300

**Published:** 2025-05-20

**Authors:** Anel Karisik, Benjamin Dejakum, Kurt Moelgg, Julian Granna, Silvia Felicetti, Raimund Pechlaner, Lukas Mayer-Suess, Thomas Toell, Lucie Buergi, Lukas Scherer, Karin Willeit, Martin Heidinger, Clemens Lang, Julia Ferrari, Stefan Krebs, Rainer Kleyhons, Heinrich Resch, Johann Willeit, Lisa Seekircher, Lena Tschiderer, Peter Willeit, Marek Sykora, Georg Schett, Wilfried Lang, Michael Knoflach, Stefan Kiechl, Christian Boehme

**Affiliations:** 1 Department of Neurology, Medical University of Innsbruck, Innsbruck, Austria; 2 VASCage-Centre on Clinical Stroke Research, Innsbruck, Austria; 3 Austrian Federal Ministry of Social Affairs, Health, Care and Consumer Protection, Vienna, Austria; 4 University of Basel, Basel, Switzerland; 5 Department of Orthopaedic Surgery and Traumatology, Clinic Donaustadt, Vienna, Austria; 6 Ludwig Boltzmann Institute Traumatology, Research Center in Cooperation with AUVA, Vienna, Austria; 7 Department of Neurology, Hospital St. John’s of God, Vienna, Austria; 8 Metabolic Bone Diseases Unit, School of Medicine, Sigmund Freud University Vienna, Vienna, Austria; 9 Institute of Clinical Epidemiology, Public Health, Health Economics, Medical Statistics and Informatics, Medical University of Innsbruck, Innsbruck, Austria; 10 Department of Public Health and Primary Care, University of Cambridge, Cambridge, UK; 11 Medical Faculty, Sigmund Freud Private University Vienna, Vienna, Austria; 12 Department of Internal Medicine 3—Rheumatology and Immunology, Friedrich-Alexander-Universität Erlangen-Nürnberg and Universitätsklinikum Erlangen, Erlangen, Germany; 13 Deutsches Zentrum Immuntherapie, Friedrich-Alexander-Universität Erlangen-Nürnberg and Universitätsklinikum Erlangen, Erlangen, Germany

**Keywords:** Ischemic stroke, prevention, fracture, mortality, transient ischemic attack, falls

## Abstract

**Background:**

Recent advances in acute stroke therapy improved short-term outcome, but some of this benefit may be lost due to post-stroke complications, including fractures.

**Aims:**

We assessed the incidence of fractures before and after stroke and transient ischemic attack (TIA), the risk factors for fractures, and the consequences for mortality, functional outcome, and quality of life.

**Methods:**

Consecutive patients with acute ischemic stroke or TIA from the prospective STROKE-CARD Registry and the randomized controlled STROKE-CARD trial and its long-term follow-up were analyzed. We prospectively assessed all fractures using self-report and documentation, records of hospitals and general practitioners, and electronic health records with all radiographs.

**Results:**

A total of 2513 patients were included (median age = 72 years (interquartile range, IQR = 61–79), 39.2% female). In the first year after the event, 145 individuals (5.8%, 95% confidence interval (CI) = 4.9%–6.7%) experienced 152 fractures corresponding to an incidence rate of 61.87 (95% CI = 52.04–71.71) per 1000 person-years. Rates were similar after stroke and TIA (60.84 and 72.28 per 1000 person-years). The incidence of fractures was more than five times higher compared to the general population (age- and sex-adjusted hazard ratio (HR) for first fracture 5.36, 95% CI = 2.49–11.52). The risk of fractures 1 year before stroke/TIA was also increased (HR = 2.99, 95% CI = 1.39–6.42). Stroke/TIA further increased the risk of fractures as documented by a comparison between fractures 1 year before and 1 year after the event (age- and sex-adjusted risk ratio = 1.69, 95% CI = 1.10–2.58). The main risk factors for fractures were falls and osteoporosis. Fracture after stroke/TIA was associated with death (adjusted odds ratio (aOR) = 2.16, 95% CI = 1.20–3.89), inability to walk (aOR = 2.06, 95% CI = 1.08–3.93), and poor quality of life.

**Conclusions:**

Patients with ischemic stroke and TIA are at high risk for future fractures. Fracture after stroke/TIA is strongly associated with death, poor functional outcome, and reduced health-related quality of life. Therefore, there is a need to incorporate fracture prevention into post-stroke care to improve patient outcomes.

**Trial Registration:**

STROKE-CARD Registry (NCT04582825, https://clinicaltrials.gov/study/NCT04582825); STROKE-CARD trial (NCT02156778, https://clinicaltrials.gov/study/NCT02156778); STROKE-CARD long-term follow-up (NCT04205006, https://clinicaltrials.gov/study/NCT04205006).

## Graphical abstract

**Graphical abstract:**
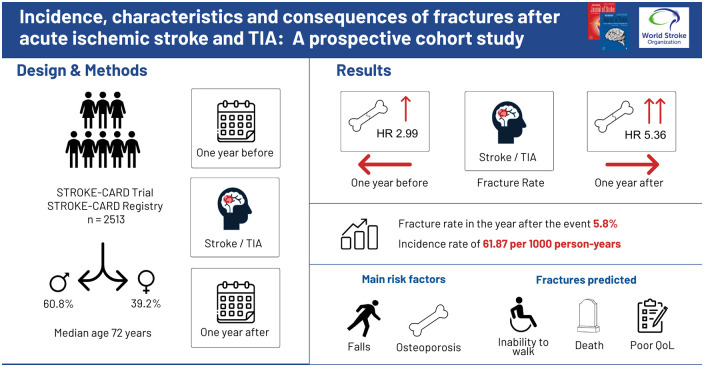

## Introduction

Ischemic stroke ranks among the primary contributors of severe disability worldwide. The current lifetime risk of ischemic stroke is 25%.^1^ There are over 12 million new ischemic strokes annually and over 100 million ischemic stroke survivors world-wide with a strong upward trajectory given the continuous aging of societies, population growth, and declining stroke mortality.^2,3^ Post-stroke complications are frequent, affect functional outcome, and jeopardize the success achieved by reperfusion therapy and stroke unit care. Fractures are a feared post-stroke complication that can affect rehabilitation and increase disability.^4–6^ Prior research determining the risk of fractures after stroke or transient ischemic attack (TIA) was mostly carried out retrospectively and reported fracture rates of 22–74 per 1000 person-years and an elevated fracture risk up to 7-fold compared to the general population in the first year after the event.^7–13^ Potential reasons for the elevated fracture risk are neurological deficits, exalted risk of falls, shared risk factors between fracture and stroke, and changes in bone structure after cerebral ischemic events termed “post-stroke osteopathy.”^14^

The aim of our study was to assess (a) the frequency of fractures before and after ischemic stroke and TIA (short and long-term), (b) risk factors for fractures, and (c), consequences on mortality, outcome, and health-related quality of life. We used combined data from two prospective studies with the same protocols and same setting. To confirm the findings, we analyzed the risk of femoral fractures 1 year after and 1 year before stroke/TIA in a nationwide population-based study, including all stroke/TIA patients in Austria occurring within three years (n = 48,996) and compared fracture risk with the general Austrian population (accompanying paper).

## Methods

### Design and participants

The study population comprises two prospective cohorts of ischemic stroke and TIA patients, the STROKE-CARD Registry (NCT04582825) and the STROKE-CARD trial (NCT02156778) with its long-term follow-up (NCT04205006). Patients were enrolled at the University Hospital Innsbruck, Austria which serves as the primary hospital for two districts of the Federal State of Tyrol and as the comprehensive stroke center for the whole state (approx. 800,000 inhabitants).

In the prospective STROKE-CARD Registry,^15^ which included a 3- and 12-month follow-up visit, we invited all surviving ischemic stroke and TIA (ABCD2-Score ⩾ 3) patients to participate. This analysis includes all patients who performed the 12-month visit before October 2023 and included 792 patients. Patients were instructed to record falls and fractures after hospital discharge and were asked for fractures 1 year before stroke/TIA.

**Figure 1.**
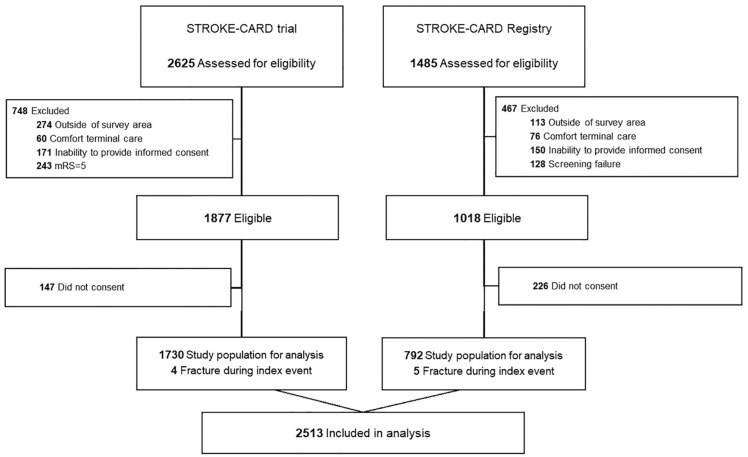
Consort flow chart of patient inclusion for STROKE-CARD trial and STROKE-CARD Registry.

The randomized STROKE-CARD trial^16^ was a pragmatic intervention trial testing effects of an intensified post-stroke disease management program on outcome and recurrence risk, not specifically tailored to prevent fractures. This analysis included all 1730 patients from study center Innsbruck (Figure 1).

Long-term follow-up was acquired for all patients with a visit either in-person or via telephone call including review of medical records between 2018 and 2021.^17^

In addition, we used the prospective Bruneck-Study to compare fracture rates of stroke/TIA patients with the general population. The Bruneck-Study is an age- and sex-stratified random sample of inhabitants of Bruneck, South Tyrol, Italy (located 70 km away from Innsbruck, Austria), with a > 93% participation rate at baseline and a thorough assessment of fractures^18^ from 1990 to 2000 in 919 individuals (details on recruitment, follow-up, and fracture ascertainment in the Bruneck-Study are provided in the supplemental material).

### Procedure and materials

Fracture ascertainment combined self-report with hospital records, GP reports and national electronic health records, including all inpatient and outpatient radiological examinations, ensuring comprehensive coverage of almost all fractures. We evaluated all in- and outpatient radiographs undertaken after the event from electronic records of all regional hospitals and the Austrian electronic health records (ELGA) containing in- and out-patient radiological examinations. In addition, medical records of the general practitioners were collected. Osteoporosis was recorded through participants’ self-report or electronic patient records at hospital admission. Quality of life at 12 months was recorded as patient-reported outcome using the EQ-5D-3L questionnaire. Modified Rankin Scale (mRS) and National Institutes of Health Stroke Scale (NIHSS) were documented by trained physicians, who had no information on fractures at admission, discharge and 12 months. Other variables were defined in the online supplement.

### Statistical analyses

All calculations were performed using SPSS version 27.0.1.0 (IBM SPSS Statistics, Armonk, NY) and R (version 4.4.2, The R Foundation for Statistical Computing, Vienna, Austria).

Pooled data were used for most analyses except for long-term fracture risk (only STROKE-CARD trial) and the comparison of fracture risk before versus after stroke/TIA (only STROKE-CARD Registry).

Depending on the underlying distribution, continuous variables are presented as mean ± standard deviation or median (IQR), and categorical variables as percentages. Univariate comparisons were performed using Pearson’s chi-square test for categorical variables and Wilcoxon Mann–Whitney U test for continuous variables. Fracture incidence was calculated for pre-stroke and post-stroke (1-year) periods, and person-time was censored at the date of death or 365 days post-stroke or TIA, whichever occurred first.

To analyze factors associated with fractures while treating death as a competing risk we employed a multivariable cause-specific hazard model with backwards elimination using the Akaike Information Criterion (AIC). Sex was forced into the model and was not subject to variable selection. We chose the cause-specific hazard model over the Fine-Gray subdistribution model, because our focus was etiological rather than prognostic, following the recommendations laid out by Lau et al.^19^

Only the first fracture event per subject was considered. Data were censored at death or end of follow-up. The proportional hazards assumption was assessed for all covariates, and time-dependent coefficients were used for variables showing significant deviations. Model estimation was conducted using the survival package in R.

We compared the incidence rates of fractures in our study population with those of a healthy cohort from the Bruneck-Study. Death and “inability to walk” (defined as mRS 4–5 vs 0–3) at 12 months were analyzed as binary outcomes using multivariable logistic regression, with fracture occurrence as the primary independent variable and adjustment for age, sex, type of event (stroke or TIA), type of study, pre-event mRS (modified Rankin Scale), inability to walk at discharge (mRS 4–5). Due to minimal missing data, a complete-case analysis was performed without imputation.

Furthermore, we compared fracture incidence rates between patients with TIA and stroke, as well as between those treated in primary versus tertiary care settings. All reported probability values are two-sided and the level of significance was set at p < 0.05.

## Results

### Frequency and characteristics of fractures

The study population (STROKE-CARD registry and trial) comprised 2513 patients (Figure 1). The STROKE-CARD Registry and trial (both recruited patients in the same setting with almost identical protocols) had similar characteristics, including median age (73 years [IQR, 62–80] vs 72 years [IQR, 61–79]), female sex (36.6% vs 40.3%), median NIHSS score (2 [IQR, 1–5] vs 2 [IQR, 1–5]), median ABCD^2^-Score (4 [IQR, 4–5] vs 4 [IQR, 3–5]), and fractures (6.4% vs 5.4%).

The median age was 72 years (IQR, 61–79) and 39.2% were female. Median NIHSS at admission and discharge was 3 (IQR, 1–6) and 1 (IQR, 0–2) (stroke patients, n = 2121), median ABCD^2^-Score at admission was 4 (IQR 4-5) (TIA patients, n = 392) (Table 1).

**Table 1.** Characteristics of patients with and without fracture 1 year after ischemic event.

	All patients (n = 2513)	Fracture (n = 145)	No fracture (n = 2368)	p value
Characteristics	No. (%) or median [IQR]			
Female sex	984 (39.2%)	69 (47.6%)	915 (38.6%)	-
Age (years)	72 (61-79)	77 (70.5-82.5)	71 (61-79)	-
Body-mass index (kg/m^2^)	26 (24-29)	25 (23-28)	26 (24-29)	0.29
Smoking	551 (21.9%)	24 (16.6%)	527 (22.3%)	0.80
Arterial hypertension	2002 (79.7%)	128 (88.3%)	1874 (79.1%)	0.40
Dyslipidemia	2200 (87.5%)	123 (84.8%)	2077 (87.7%)	0.33
Diabetes	481 (19.1%)	35 (24.1%)	446 (18.8%)	0.17
Atrial fibrillation	587 (23.4%)	51 (35.2%)	536 (22.6%)	0.17
Peripheral artery disease	205 (8.2%)	17 (11.7%)	188 (7.9%)	0.16
History of ischemic stroke or TIA	593 (23.6%)	40 (27.6%)	553 (23.4%)	0.58
Heart failure	196 (7.8%)	19 (13.1%)	177 (7.5%)	0.21
Osteoporosis	199 (7.9%)	34 (23.4%)	165 (7.0%)	<0.001
Charlson Comorbidity Index^a^	1 [0-1]	1 [0-2]	1 [0-1]	0.001

p values (derived from Wald tests) for differences between patients with and without fractures, adjusted for age and sex using logistic regression models.

a Before index event.

Abbreviation: TIA: transient ischemic attack.

In total, 145 individuals (5.8% [95% CI = 4.9%–6.7%]) experienced 152 fracture events in the year after the ischemic event with an incidence of fractures of 61.87 per 1000 person-years (95% CI = 52.04–71.71). The risk was similar between stroke and TIA (60.84 [95% CI = 51.15–72.39] vs 72.28 [95% CI = 41.70–108.42] per 1000 person-years; p = 0.52), in primary and tertiary referral patients (66.88 [95% CI = 56.62–77.92] vs 50.9 [95% CI = 39.5–66.5] per 1000 person-years; p = 0.15) and highest in patients with falls after the event (206.70 per 1000 person-years [95% CI = 167.35–246.05]) and osteoporosis (179.32 per 1000 person-years [95% CI = 119.91–238.73]).

Fractures affected the femoral neck (16.6%) and other lower limbs (17.9%; in total 34.5%), upper limbs (21.4%), thorax (19.3%), spine (16.6%), and skull (8.3%). One out of four patients experienced multiple fractured bones (n = 38, 26.2%).

**Figure 2.**
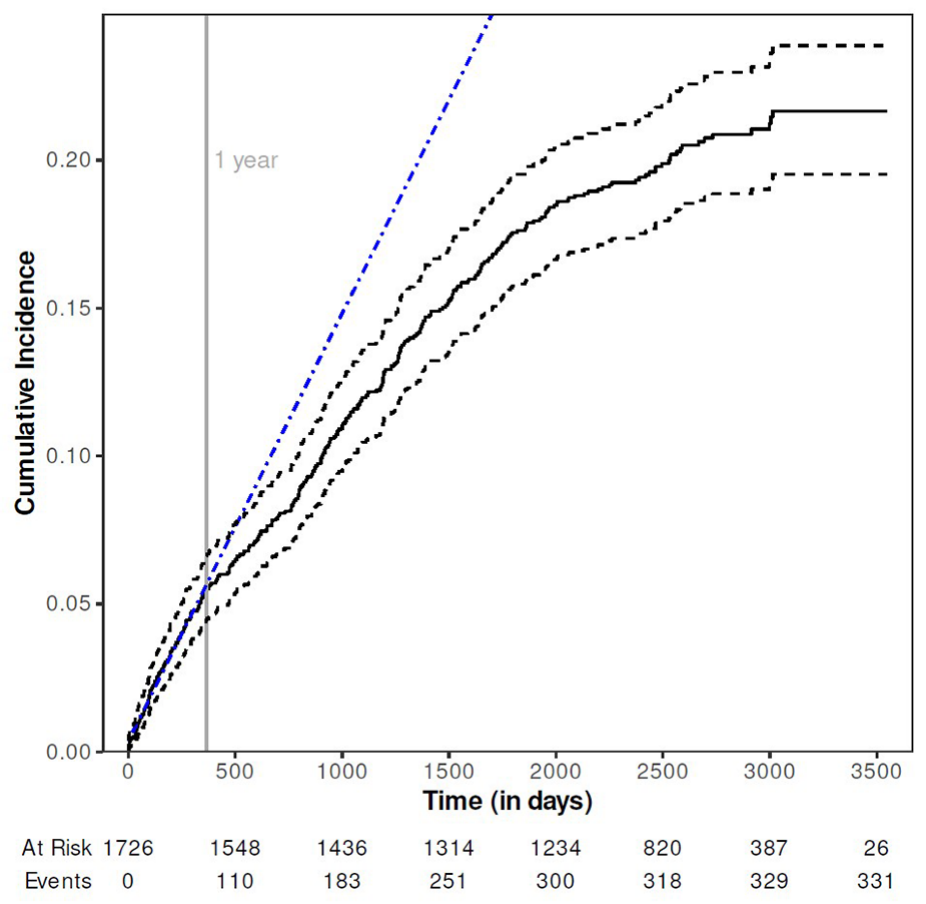
Kaplan–Meier curve of the cumulative incidence of fracture risk after ischemic stroke or TIA. Solid line shows the cumulative incidence of fracture after ischemic stroke or TIA with dashed lines indicating 95% CI; the dashed-dotted blue line represents a linear prognosis of fracture risk based on the event rate in the period from 0 to 365 days.

In the long-term follow-up of the STROKE-CARD trial, 385 patients experienced 483 fracture events (follow-up after index event, median 5.0 years), whereby the risk gradually declined over time (Figure 2).

In the STROKE-CARD Registry, 32 patients (4.1%) had at least one fracture event in the year prior to the index event (40.70 per 1000 person-years [95% CI = 27.76–45.18]) and 54 patients (6.9%) in the year after the event (70.80 per 1000 person-years [95% CI = 53.22–92.34], age-/sex-adjusted relative risk 1.69 [95% CI = 1.10–2.58], p = 0.02). Both rates are elevated in comparison with the general population in the Bruneck-Study (age-/sex-adjusted hazard ratio [HR] 2.99 [95% CI = 1.39–6.42] and 5.36 [95% CI = 2.49–11.52]). When considering post-stroke/TIA fractures of the STROKE-CARD trial as well, the age-/sex-adjusted HR compared to the general population was similar at 5.38 (95% CI = 2.35–12.31).

### Risk factors of fractures

Comparing characteristics of patients with/without fractures 1 year after the event, those with fractures were more often female (47.6% vs 38.6%, p = 0.02) and of higher age (median, 77 vs 71 years, p < 0.001). Patients with fractures had more falls after the event (71.0% vs 17.4%, p < 0.001), higher prevalence of osteoporosis (23.4% vs 7.0%, p = 0.001) and more comorbidities (Charlson Comorbidity Index (CCI) 1 [IQR, 0–2] vs 1 [IQR, 0–1], p = 0.001).

There was no association between stroke etiology and localization with fracture risk and neither between side of fractures and side of stroke (Table S1 and Table S2). Concerning neurological deficits in stroke patients, facial palsy, leg palsy, dysarthria, ataxia, and reduced level of consciousness were associated with fractures (Table S3). Also, higher discharge NIHSS and mRS were associated with fracture risk (Table S1). Patients with fractures were significantly more often prescribed proton-pump inhibitors, insulin and neuropsychiatric drugs (Table S4).

In a multivariable cause-specific hazard model, falls after the event and osteoporosis were the most prominent risk factors with cause-specific hazard in alive subjects, followed by age, comorbidity, and mRS at discharge (Table 2). The significant association with medication was lost when adjusting for post-event falls and osteoporosis. Long-term risk factors for fractures are displayed in Table S5.

**Table 2.** Factors associated with fracture 1 year after stroke and TIA.

Characteristics	HR [CI]	p value
Age (per 1 year)	1.02 [1.00-1.03]	0.026
Sex		
Male	—	
Female	1.11 [0.79-1.57]	0.54
Falls post stroke		
No	—	
Yes	8.13 [5.62-11.80]	<0.001
Osteoporosis		
No	—	
Yes	2.49 [1.66-3.72]	<0.001
mRS at discharge		
0	—	
1	1.04 [0.58-1.84]	0.90
2	0.86 [0.51-1.45]	0.57
3	1.79 [1.08-2.95]	0.023
4 and 5	1.71 [0.97-3.00]	0.063
Charlson Comorbidity Index (per 1 score point)	1.14 [1.03-1.27]	0.015

Hazard ratios (HR) and 95% confidence intervals (CIs) were calculated by multivariable cause-specific hazard model regression with backward elimination. Sex was excluded from variable selection to ensure inclusion in the final model. Variable candidates: age, body-mass index, Charlson Comorbidity Index, falls post stroke, modified Rankin Scale at discharge, insulin, neuropsychiatric drugs, NIHSS, osteoporosis, proton-pump inhibitors, type of study, sum of medication, type of stroke event (stroke/TIA).

Abbreviations: HR, hazard ratio; CI, confidence interval; mRS, modified Rankin Scale.

### Outcome of patients after fracture

In 96.7% of the 2411 surviving patients, health-related quality-of-life at 12-month follow-up was available. Across all five dimensions, level two or three (indicating mild or severe problems) were significantly more often reported by patients with fractures with the strongest association observed for “usual activities” and “self-care” (Table S6). In 98.8% of the 2411 surviving patients at 12 months, mRS was available. Post-stroke/TIA fractures are strongly associated with death (11.7% vs 3.6%; median time after fracture 81 days [IQR, 29–147]) and inability to walk (mRS, 4–5) (16.4% vs 5.0%) with an adjusted odds ratio (OR) of 2.07 (95% CI = 1.14–3.73) and 2.08 (95% CI = 1.09–3.95) (Table S6).

## Discussion

### High fracture risk in stroke and TIA patients

Our study indicates that ischemic stroke and TIA patients are at very high risk of fractures (incidence in the first year after the event, 61.87 per 1000 person-years). This is in line with a recent study from Australia^7^ (incidence, 74 per 1000 person-years) but exceeds estimates from previous studies (22–41 per 1000 person-years) (Table S7). Possibly, the high incidence of fractures in our cohort is due to the prospective study design with a thorough assessment of fractures which is difficult to obtain in retrospect. The highest fracture rates in our study were observed in patients with falls after event (206.70 per 1000 person-years) and osteoporosis (179.32 per 1000 person-years). The fracture risk in our study exceeds the risk of the general population five-fold. Patients with Parkinson’s disease have an incidence of fractures of 33.9 per 1000 person-years, 1.5 times higher than the general population^20^ and patients with osteoporosis have a 1-year incidence rate of osteoporotic fractures of hip, humerus or distal forearm of 17.9–36.7 per 1000 person-years.^21^

In the long run, the incidence of fractures in patients with stroke and TIA gradually decreases (Figure 2). There are few studies on long-term risk of fractures which support our findings.^8,9,11,22,23^ Similarly, fracture risk after an osteoporotic vertebral fracture was highest in the first year and substantially lower afterwards.^24^

### Contributors of the high fracture risk

Patients with osteoporosis are at increased risk of stroke, indicating that environmental and maybe genetic risk factors as well as comorbidity of osteoporosis, fractures, atherosclerosis and stroke are partly shared.^17,25^ Our study supports this hypothesis as the risk of fractures 1 year before stroke/TIA was elevated compared to the general population and CCI was part of our prediction model.

Importantly, stroke/TIA further elevate the risk of fractures as documented by a comparison between fracture rate 1 year before and 1 year after the event (relative risk 1.69) which is consistent with Austrian nationwide analyses (accompanying paper) and an increase of fracture incidence from 45 pre-event to 74 post-event per 1000 person-years in the recent study by Dalli et al.^7^ Further research is needed to disentangle this relationship.

There are several potential and disputable contributors for this enhanced fracture risk.^7^ First, neurological deficits after stroke may result in falls and fractures. However, the absolute risk of fractures in previous studies^7,22^ and also in our study was similar between stroke and TIA and there is no correlation between side of fracture and side of stroke. Therefore, neurological deficits may not be the key driver of fractures although patients with a discharge mRS ⩾ 3 are associated with higher cause-specific hazard (Table 2). Second, we found that neuropsychiatric drugs, insulin, and proton-pump inhibitors increased fracture risk (Table S4). Significance of these associations is lost when we additionally adjusted for post-event falls and osteoporosis. There is compelling evidence that neuropsychiatric drugs including neuroleptics and benzodiazepines increase the risk of falls and fractures and insulin reflects comorbidities in diabetic patients. Proton-pump inhibitors might have an effect on bone metabolism or might be a bystander of polypharmacy.^26^ Third, falls after stroke are the most important risk factor for fracture risk (71.0% of patients with fracture vs 17.4%). Stroke patients have an up to 3-fold increased risk of falls due to impaired balance, gait abnormality, fatigue, vertigo, sedative and psychotropic medication, cognitive impairment, syncope, visual impairment and pain.^27^ Fourth, experimental studies showed that stroke *per se* affects bone quality. Bone remodeling is controlled by the autonomic nervous system^28^ and the stress response after stroke/TIA activates ß_2_-adrenergic signaling to osteoblasts, inhibiting osteoblast proliferation and induce release of RANKL which augments osteoclastogenesis and bone resorption.^29^ In addition, stroke activates hematopoietic stem cells via increased ß_3_-adrenergic sympathetic tone, leading to higher output of inflammatory monocytes and neutrophils from the bone marrow, which triggers inflammation and impairs bone quality.^30^ Recent pilot studies also reported trabecular loss after stroke.^31^

### Outcome of patients after fracture

One previous study indicated that fractures decrease the health-related quality-of-life in patients with stroke/TIA (after 90–180 days) but specifies the limitation that the EQ-5D-3L was only available for a subset of patients.^7^ In our study, the EQ-5D-3L utility score was completed in 96.7% of surviving patients at 12 months. Post-stroke fractures were associated with a pronounced deterioration of quality-of-life with more problems in self-care, mobility, usual activities, pain/discomfort, and anxiety/depression. To our knowledge, this is the first study that showed a substantially increased risk of mortality (aOR = 2.07) and disability (aOR = 2.08) in stroke/TIA patients with any fractures (Table S6). Previous reports focusing on femoral fractures yielded controversial results.^6^

### Clinical implications

Our study suggests that stroke/TIA patients have a very high risk of fractures, which should stimulate rigorous prevention of fractures. Multifactorial and specific interventions including exercise, intensified gait/balance and fall training can reduce the fall risk by about 25%.^27,32^ It is recommended to avoid drugs that increase fractures such as insulin sensitizers (e.g. pioglitazone), Vitamin K antagonists, and non-mandatory psychotropic drugs like neuroleptics and benzodiazepines. Ischemic stroke/TIA patients might benefit from an osteoporosis screening and early treatment when indicated.^14^ Screening for fragility fractures using the Fracture Risk Assessment Tool (FRAX) showed to be predictive for femur fractures in stroke patients. In the Ontario Stroke Registry, a scoring system for stroke/TIA patients was developed ranging from −9 to +36 points and predicted a risk-range of fragility fractures from 0.5 to 34.1%.^33^ Prevention of fractures independent of stroke is successful. For example, in Norway, age-adjusted hip fracture-incidence decreased by 27% (1999–2019) and was partly attributed to reduction in smoking (explained 11%), increased physical activity (16%), avoidance of benzodiazepines (13%), and osteoporotic treatment (21%).^34^

### Limitations and strengths

Our cohorts were conducted in Europe and >99% of all patients were of European descent. Therefore, the results cannot be easily transferred to low-middle income countries and other regions without universal healthcare systems. For a comparison with the general population, we used the Bruneck-Study with a thorough assessment of fractures between 1990 and 2000^18^ prior to the STROKE-CARD studies (2014–2023). However, age- and sex-standardized fracture rates declined over the last decades due to prevention, novel therapies and protectors in sports/traffic, so that our comparison of fracture rates between the stroke/TIA cohort and the general population may be conservative.^34^ The estimates of fracture risk in stroke/TIA patients are conservative as well, partly because we did not include fractures occurring concurrent with stroke/TIA and several exclusion criteria of our studies (terminal illness, drug/alcohol abuse) are linked to high fracture risk. To improve accuracy and minimize recall bias, fracture ascertainment combined self-report with hospital records, GP reports and national electronic health records. This comprehensive approach ensured robust data collection, although there may have been some under-recording of patients who died before follow-up. Although a small proportion of eligible patients were excluded from the study (terminal illness 2%, substance abuse 1%, and severe disability 9%), the inclusion of a broad stroke and TIA population from two cohorts (92% and 77% of all stroke patient admitted to the hospital, Figure 1) strengthens the generalizability of our findings.

## Conclusion

Patients with ischemic stroke/TIA are high-risk patients for fractures. Post-stroke fractures substantially worsen health-related quality of life after 12 months and are strongly associated with death and disability. Stroke physicians should be aware of this frequent post-stroke complication. Therefore, there is a need for inclusion of fracture prevention in post-stroke care to enhance functional outcome and quality of life after stroke/TIA.

## Supplemental Material

sj-docx-1-wso-10.1177_17474930251345300 – Supplemental material for Incidence, characteristics, and consequences of fractures after acute ischemic stroke and TIA—A prospective cohort study Supplemental material, sj-docx-1-wso-10.1177_17474930251345300 for Incidence, characteristics, and consequences of fractures after acute ischemic stroke and TIA—A prospective cohort study by Anel Karisik, Benjamin Dejakum, Kurt Moelgg, Julian Granna, Silvia Felicetti, Raimund Pechlaner, Lukas Mayer-Suess, Thomas Toell, Lucie Buergi, Lukas Scherer, Karin Willeit, Martin Heidinger, Clemens Lang, Julia Ferrari, Stefan Krebs, Rainer Kleyhons, Heinrich Resch, Johann Willeit, Lisa Seekircher, Lena Tschiderer, Peter Willeit, Marek Sykora, Georg Schett, Wilfried Lang, Michael Knoflach, Stefan Kiechl and Christian Boehme in International Journal of Stroke
