# Supplementary material for: Incidence, characteristics, and consequences of fractures after acute ischemic stroke and TIA—A prospective cohort study
Source: Int J Stroke. 2025 May 20;20(9):1141–9. doi: 10.1177/17474930251345300 (PMC12521769; doi:10.1177/17474930251345300)
Supplement: sj-docx-1-wso-10.1177_17474930251345300 – Supplemental material for Incidence, characteristics, and consequences of fractures after acute ischemic stroke and TIA—A prospective cohort study [file sj-docx-1-wso-10.1177_17474930251345300.docx]

**Supplementary Online Content**

**Incidence, characteristics and consequences of fractures after acute ischemic stroke and TIA – a prospective cohort study**

**Content**

Supplemental Methods

Supplemental Tables

Table S1. Localization, etiology and outcome measures of index-event in patients with and without fracture.

Table S2. Association between side of stroke/TIA and fracture side.

Table S3. Persistent disabilities at discharge in patients with and without fracture.

Table S4. Medication at discharge of patients with and without fracture.

Table S5. Predictors of fracture risk in long-term follow-up.

Table S6. Consequences of fractures post stroke or TIA.

Table S7. Summary of publications on fracture risk after stroke and TIA.

**Supplemental Methods**

All types of fractures were included except those derived from falls caused directly by acute stroke/TIA (n=9) and information on localization, date and etiology were recorded. Fracture events with multiple fractured bones (e.g. rib series fractures) were counted as single fracture event. Stroke/TIA etiology was categorized in accordance with the TOAST criteria. Body regions were defined as upper limb (fingers to humerus), lower limb (toes to pelvis, except femoral neck), femoral neck, spine (coccyx to C1 vertebra), skull, and thorax (including scapula, clavicle, ribs and sternum). Fracture side was defined as left, right, both and trunk (here including coccyx to C1 vertebra and sternum).

Body mass index was calculated as weight in kilograms divided by the square of height in meters. Smoking status was recorded using participants’ self-report. Dyslipidemia was defined as LDL-Cholesterol > 100 mg/dl or HDL-cholesterol < 40 mg/dl or total cholesterol > 200 mg/dl or lipoprotein(a) > 75 nmol/l or triglycerides > 150 mg/dl in the initial blood sampling at initial ischemic stroke-event related hospitalization or if documented in electronic patient history. Arterial hypertension, atrial fibrillation, diabetes mellitus, cerebrovascular history (ischemic stroke or TIA), peripheral artery disease and heart failure were recorded through participants’ self-report or electronic patient history and through in-hospital assessment. Falls after the event were recorded using patients’ self-report and records. The location of the cerebral ischemic lesion/TIA was classified as anterior (brain regions with vascular supply of anterior or middle cerebral artery) or posterior circulation (brain regions with vascular supply of posterior, vertebral or basilar artery) and both.

STROKE-CARD Registry: After the STROKE-CARD trial showed beneficial effects by reducing cardiovascular events and improving health-related quality of life, the program was implemented as a standard of care in December 2020 by the Department of Neurology at the Medical University of Innsbruck and the St. John of God Hospital in Vienna including a 3- and 12-month follow-up outpatient visit after the index event. Data of stroke and TIA patients were collected prospectively in the STROKE-CARD Registry which also includes patients with severe disability (mRS=5) at discharge. For the present analysis, data collected at the study center Innsbruck was used. Patient history of fractures one year before index event was collected using a patient survey, the clinical information system of the University Hospital Innsbruck (KIS-comprehensive database system, that allows hospital staff to document measures and collect patient information (diagnoses, new and previous findings, new results, etc.), Austrian electronic health records (ELGA-database of all available health related data in Austria from health service providers, i.e. hospitals, practitioners and pharmacies; implemented in 2015 in hospitals and 2019 for practitioners) and health records from the respective general practitioners.

STROKE-CARD trial: Randomized open pragmatic intervention trial testing effects of an intensified post-stroke disease management program on 1-year patient outcome (no predefined focus was set on post-stroke fractures). For the present analysis, data on patients collected at the study center Innsbruck is utilized. Patients with acute ischemic stroke or TIA admitted to the University Hospital Innsbruck between January 2014 and December 2017 were allocated to STROKE-CARD care or standard care in a 2:1 randomization. STROKE-CARD care involved an additional outpatient appointment scheduled three months after the index event including a comprehensive re-evaluation focusing on post-stroke management (e.g. residual deficits, post-stroke complications, risk factor control).

STROKE-CARD long-term follow-up: Patients enrolled in the STROKE-CARD trial at the study center Innsbruck, Austria, were invited to an additional long-term follow-up visit either in person or via telephone call between December 2019 and November 2021. Long-term follow-up fracture data was acquired for all patients, who were included in the STROKE-CARD trial.

Bruneck Study: Community-based cohort study in the city of Bruneck, South Tyrol, Italy. A sex- and age-stratified random sample of all residents living in the city of Bruneck, aged 40-79 years, was recruited in 1990 (125 women and 125 men in each decade of age; n = 1000). After baseline examination in 1990, follow-up examinations were conducted at 5-year intervals. A thorough assessment of fractures was performed from 1990 to 2000 in 919 individuals. This included reviewing all radiographs of patients in the catchment area, as the Bruneck hospital is the only provider of radiographs in the region during the study interval.

**Supplemental Tables**

**Table S1. Localization, etiology and outcome measures of index-event in patients with and without fracture.**

|  | **All patients** | **Fracture** | **No fracture** | **P value** |
| --- | --- | --- | --- | --- |
|  | No. (%) or mean [±SD] or median [IQR] | | |  |
| **Index event – Ischemic stroke (n=2121)** |  |  |  |  |
| Stroke territory |  |  |  |  |
| Anterior circulation | 1236 (58.3%) | 70 (57.9%) | 1166 (58.3%) | .48 |
| Posterior circulation | 669 (31.5%) | 39 (32.2%) | 630 (31.5%) | .56 |
| Both | 216 (10.2%) | 12 (9.9%) | 204 (10.2%) | .80 |
| Lesion localization |  |  |  |  |
| Unilateral left | 967 (45.6%) | 59 (48.8%) | 908 (45.4%) | .57 |
| Unilateral right | 922 (43.5%) | 47 (38.8%) | 875 (43.8%) | .23 |
| Bilateral lesion | 232 (10.9%) | 15 (12.4%) | 217 (10.9%) | .30 |
| Stroke severity |  |  |  |  |
| NIHSS, admission | 3 [1-6] | 4 [2-8] | 3 [1-6] | .047 |
| mRS, admission | 3 [2-4] | 3 [2-4] | 3 [2-4] | <.001 |
| NIHSS, discharge | 1 [0-2] | 2 [0-4] | 1 [0-2] | <.001 |
| mRS, discharge | 2 [1-3] | 3 [1-3] | 2 [1-2] | <.001 |
| **Index event – TIA (n=392)** |  |  |  |  |
| Vascular territory |  |  |  |  |
| Anterior circulation | 263 (67.1%) | 16 (66.7%) | 247 (67.1%) | .95 |
| Posterior circulation | 129 (32.9%) | 8 (33.3%) | 121 (32.9%) | .95 |
| Symptomatic region |  |  |  |  |
| Left cerebral hemisphere | 164 (41.8%) | 13 (54.2%) | 151 (41.0%) | .27 |
| Right cerebral hemisphere | 113 (28.8%) | 5 (20.8%) | 108 (29.3) | .47 |
| Brainstem/Basilar territory | 115 (29.3%) | 6 (25.0%) | 109 (29.6%) | .62 |
| TIA risk level |  |  |  |  |
| ABCD2 Score | 4.3 [±1.0] | 4.4 [±0.9] | 4.3 [±1.0] | .97 |
| **Etiology of index event (n=2513)** |  |  |  |  |
| TOAST criteria |  |  |  | .50 |
| Large-artery arteriosclerosis | 496 (19.7%) | 19 (13.1%) | 477 (20.1%) | .06 |
| Cardio embolism | 611 (24.3%) | 48 (33.1%) | 563 (23.8%) | .32 |
| Small-artery occlusion | 524 (20.9%) | 35 (24.1%) | 489 (20.7%) | .47 |
| Other determined etiology | 108 (4.3%) | 4 (2.8%) | 104 (4.4%) | .97 |
| Undetermined etiology | 774 (30.8%) | 39 (26.9%) | 735 (31.0%) | .97 |

P values for differences between patients with and without fractures, adjusted for age and sex using logistic regression models. P values were derived from Wald tests.

Abbreviations: mRS, modified Rankin Scale; NIHSS, National Institutes of Health Stroke Scale; TIA, transient ischemic attack.

**Table S2. Association between side of stroke/TIA and fracture side.**

|  | **All limb fractures**  **(n=109)** | **Right-sided fracture** | **Left-sided fracture** | **P value** |
| --- | --- | --- | --- | --- |
| **Lesion localization** |  |  |  | .55 |
| Unilateral left | 61 (56.0%) | 31 (54.4%) | 30 (57.7%) |  |
| Unilateral right | 35 (32.1%) | 18 (31.6%) | 17 (32.7%) |  |
| Bilateral or brainstem | 13 (11.9%) | 8 (14.0%) | 5 (9.6%) |  |

P values for differences between patients with right-sided and left-sided limb fracture, adjusted for age and sex using logistic regression models. P values were derived from Wald tests.

**Table S3. Persistent disabilities at discharge in patients with and without fracture.**

|  | **All patients** (n=2513) | **Fracture**  (n=145) | **No fracture**  (n=2368) | **P value** |
| --- | --- | --- | --- | --- |
| **Neurological deficits at discharge** |  |  |  |  |
| NIHSS sub-items |  |  |  |  |
| Reduced level of consciousness | 78 (3.7%) | 10 (8.3%) | 68 (3.4%) | .042 |
| Gaze palsy | 65 (3.1%) | 3 (2.5%) | 62 (3.1%) | .56 |
| Hemianopsia | 214 (10.1%) | 13 (10.7%) | 201 (10.1%) | .94 |
| Facial palsy | 472 (22.3%) | 40 (33.1%) | 432 (21.6%) | .019 |
| Arm palsy | 405 (19.1%) | 33 (27.3%) | 372 (18.6%) | .090 |
| Leg palsy | 283 (13.3%) | 27 (22.3%) | 256 (12.8%) | .020 |
| Limb ataxia | 199 (9.4%) | 18 (14.9%) | 181 (9.0%) | .040 |
| Sensory deficit | 281 (13.2%) | 18 (14.9%) | 263 (13.2%) | .45 |
| Aphasia | 229 (10.8%) | 12 (9.9%) | 217 (10.9%) | .57 |
| Dysarthria | 275 (13.0%) | 28 (23.1%) | 247 (12.4%) | .006 |
| Neglect | 94 (4.4%) | 10 (8.3%) | 84 (4.2%) | .06 |

P values for differences between patients with and without fractures, adjusted for age and sex using logistic regression models. P values were derived from Wald tests.

Abbreviations: NIHSS, National Institutes of Health Stroke Scale.

**Table S4. Medication at discharge of patients with and without fracture.**

|  | **All patients** (n=2513) | **Fracture**  (n=145) | **No fracture**  (n=2368) | **P value** |
| --- | --- | --- | --- | --- |
| **Discharge medications** |  |  |  |  |
| **Coagulation drugs** |  |  |  |  |
| Antiplatelet drugs | 1650 (65.7%) | 86 (59.3%) | 1564 (66.0%) | .97 |
| Vitamin K antagonists | 71 (2.8%) | 3 (2.1%) | 68 (2.9%) | .80 |
| Oral anticoagulants | 519 (20.7%) | 39 (26.9%) | 480 (20.3%) | .55 |
| **Antihypertensive drugs** |  |  |  |  |
| AT-II antagonists | 621 (24.7%) | 37 (25.5%) | 584 (24.7%) | .91 |
| ACE inhibitors | 358 (14.2%) | 19 (13.1%) | 339 (14.3%) | .77 |
| Diuretics | 569 (22.6%) | 44 (30.3%) | 525 (22.2%) | .22 |
| Beta-Blockers | 585 (23.3%) | 37 (25.5%) | 548 (23.1%) | .97 |
| Calcium channel blockers | 644 (25.6%) | 46 (31.7%) | 598 (25.3%) | .20 |
| **Lipid lowering drugs** |  |  |  |  |
| Statins | 1754 (69.8%) | 104 (71.7%) | 1650 (69.7%) | .40 |
| Ezetimibe | 227 (9.0%) | 16 (11.0%) | 211 (8.9%) | .21 |
| PCSK9 inhibitors | 17 (0.7%) | 0 (0.0%) | 17 (0.7%) | .99 |
| **Neuropsychiatric drugs** |  |  |  |  |
| Anti-seizure drugs | 117 (4.7%) | 12 (8.3%) | 105 (4.4%) | .049 |
| Neuroleptics | 111 (4.4%) | 13 (9.0%) | 98 (4.1%) | .026 |
| Antidepressants | 292 (11.6%) | 28 (19.3%) | 264 (11.1%) | .019 |
| Benzodiazepines | 206 (8.2%) | 23 (15.9%) | 183 (7.7%) | .027 |
| **Diabetic drugs** |  |  |  |  |
| Oral antidiabetics | 245 (9.8%) | 15 (10.3%) | 231 (9.8%) | .86 |
| Insulin and analogs | 83 (3.3%) | 11 (7.6%) | 72 (3.0%) | .004 |
| **Others** |  |  |  |  |
| Levothyroxine | 422 (16.8%) | 25 (17.2%) | 297 (16.8%) | .53 |
| Proton pump inhibitors | 737 (29.3%) | 57 (39.3%) | 680 (28.7%) | .028 |
| Corticosteroids | 77 (3.1%) | 4 (2.8%) | 73 (3.1%) | .80 |
| Osteoporosis drugs | 260 (10.3%) | 19 (13.1%) | 241 (10.2%) | .96 |

P values for differences between patients with and without fractures, adjusted for age and sex using logistic regression models. P values were derived from Wald tests. Osteoporosis drugs: Alendronate, Risedronate, Ibandronate, Zoledronic acid, Vitamin D, Calcium.

**Table S5. Factors associated with fracture risk in long-term follow-up.**

| **Characteristic** | **HR** [CI] | **P value** |
| --- | --- | --- |
| Age (per one year) | 1.02 [1.0-1.0] | <.001 |
| Sex |  | <.001 |
| male | — |  |
| female | 1.46 [1.2-1.8] |  |
| Osteoporosis |  | <.001 |
| no | — |  |
| yes | 2.81 [2.2-3.6] |  |
| Body-mass index | 0.98 [0.96-1.01] | 0.19 |
| Hypertension |  | 0.11 |
| yes | — |  |
| no | 0.77 [0.6-1.1] |  |
| Falls post stroke |  | <.001 |
| yes (year 1) | 6.74 [4.7-9.7] |  |
| yes (year 2) | 1.50 [0.8-2.8] |  |
| yes (> year 2) | 1.75 [1.3-2.5] |  |
| CCI |  | .04 |
| CCI (year 1) | 1.10 [1.0-1.2] |  |
| CCI (year 2) | 1.23 [1.0-1.5] |  |
| CCI (> year 2) | 1.00 [0.9-1.1] |  |
| mRS >= 3 |  | .003 |
| yes (year 1) | 1.80 [1.3-2.5] |  |
| yes (year 2) | 1.19 [0.6-2.3] |  |
| yes (> year 2) | 0.74 [0.5-1.1] |  |

Hazard ratios (HR) and 95% confidence intervals (CI) were calculated by multivariable cause-specific hazard model.

Abbreviations: mRS, modified Rankin Scale; CCI, Charlson Comorbidity Index.

**Table S6. Consequences of fractures post stroke or TIA.**

|  | **All patients** | **Fracture** | **No fracture** | **P value** | **OR** [CI] | **adjusted OR** [CI] |
| --- | --- | --- | --- | --- | --- | --- |
| **EQ5D3L at 12-months** (n=2331) | | | | | | |
| **Mobility** |  |  |  | <.001 | 2.74 [1.90-3.95] | 1.66 [1.08-2.54] |
| 1 “no problems” | 1559 (66.9%) | 54 (43.9%) | 1505 (68.2%) |  |  |  |
| 2 “mild problems” | 664 (28.5%) | 58 (47.2%) | 606 (27.4%) |  |  |  |
| 3 “severe problems” | 108 (4.6%) | 11 (8.9%) | 97 (4.4%) |  |  |  |
| **Self-care** |  |  |  | <.001 | 3.02 [2.09-4.36] | 1.88 [1.21-2.92] |
| 1 “no problems” | 1756 (75.3%) | 64 (52.0%) | 1692 (76.6%) |  |  |  |
| 2 “mild problems” | 361 (15.5%) | 37 (30.1%) | 324 (14.7%) |  |  |  |
| 3 “severe problems” | 214 (9.2%) | 22 (17.9%) | 192 (8.7%) |  |  |  |
| **Usual activities** |  |  |  | <.001 | 3.85 [2.63-5.64] | 2.62 [1.70-4.06] |
| 1 “no problems” | 1532 (65.7%) | 43 (35.0%) | 1489 (67.4%) |  |  |  |
| 2 “mild problems” | 532 (22.8%) | 548 (39.0%) | 484 (21.9%) |  |  |  |
| 3 “severe problems” | 267 (11.5%) | 32 (26.0%) | 235 (10.6%) |  |  |  |
| **Pain/discomfort** |  |  |  | .007 | 1.69 [1.17-2.43] | 1.32 [0.91-1.93] |
| 1 “no problems” | 1475 (63.3%) | 63 (51.2%) | 1412 (63.9%) |  |  |  |
| 2 “mild problems” | 716 (30.7%) | 47 (38.2%) | 669 (30.3%) |  |  |  |
| 3 “severe problems” | 140 (6.0%) | 13 (10.6%) | 127 (5.8%) |  |  |  |
| **Anxiety/depression** |  |  |  | .003 | 1.79 [1.23-2.61] | 1.52 [1.02-2.24] |
| 1 “no problems” | 1717 (73.7%) | 76 (61.8%) | 1641 (74.3%) |  |  |  |
| 2 “mild problems” | 489 (21.0%) | 34 (27.6%) | 455 (20.6%) |  |  |  |
| 3 “severe problems” | 125 (5.4%) | 13 (10.6%) | 112 (5.1%) |  |  |  |
|  | | | | | | |
| **Other outcome measures at 12-months** | | | | | | |
| **Death within 12-months after ischemic event** (n=2513) | | | | <.001 | 3.40 [1.97-5.89] | 2.07 [1.14-3.73] |
| No | 2411 (95.9%) | 128 (88.3%) | 2283 (96.4%) |  |  |  |
| Yes | 102 (4.1%) | 17 (11.7%) | 85 (3.6%) |  |  |  |
| **Inability to walk at 12-months after ischemic event** (n=2383^a^) | | | | <.001 | 3.72 [2.25-6.16] | 2.08 [1.09-3.95] |
| mRS ≤ 3 | 2249 (94.4%) | 107 (83.6%) | 2142 (95.0%) |  |  |  |
| mRS = 4 and 5 | 134 (5.6%) | 21 (16.4%) | 113 (5.0%) |  |  |  |

Unadjusted P values (derived from Wald tests) for differences between patients with and without fractures. Risk of event (i.e. reported problem in EQ5D3L subdomain at 12-months, death or inability to walk at 12 months) is presented as unadjusted odds ratio (OR) or adjusted for age, sex, type of event (stroke or TIA), type of study, pre-event mRS (modified Rankin Scale), inability to walk at discharge (mRS 4- 5) using binary logistic regression.

^a^ excluding 102 patients who died within 1 year and 28 patients with unknown mRS at follow-up.

Abbreviations: OR, odds ratio; CI, confidence interval

**Table S7. Summary of publications on fracture risk after stroke and TIA.**

| **Publication** | **Type of cohort** | **Region / country** | **Number of patients** | **Follow-up (up to) in years** | **Fracture risk after ischemic event** | **Fracture risk before ischemic event** | **TIA patients included** | **Fracture risk increasing / reducing factors** |
| --- | --- | --- | --- | --- | --- | --- | --- | --- |
| Ramnemark et al., Osteoporos Int. 1998^1^ | RC | Sweden | 1139 (9% hemorrhagic stroke) | 10 | 2-4-fold risk (hip fractures) (confidence interval [CI] n.a.) Incidence of 37 per 1000 person-years [CI n.a.] | n.a. | yes | older age ↑, female sex ↑ |
| Kanis et al., Osteoporos Int., 1999^2^ | RC | Europe | 730 (men only, % of hemorrhagic strokes n.a.) | n.a. | RR 2.42 - stroke with hemiplegia (all fractures) [95%CI 1.5–3.9, *P*<.001] | n.a. | yes | hemiplegia after ischemic event ↑ |
| Ramnemark et al., Stroke, 2000^3^ | RC | Sweden | 1545 (% of hemorrhagic strokes n.a.) | 5 | n.a. | n.a. | no | n.a. |
| Melton et al., Osteoporos Int., 2001^4^ | RC | USA | 387 (ischemic stroke only) | 25 | 2-fold risk (hip fracture) [95%CI 1.5–2.9] Incidence of 74 per 1000 person-years [CI n.a.] | n.a. | no | severe functional impairment ↓ |
| Kanis et al., Stroke, 2001^5^ | PC | United Kingdom | 273288 (% of hemorrhagic strokes n.a.) | 10 | 7-fold risk (all fractures within the first year) [CI n.a.] | n.a. | no | duration of hospital stay ↑, younger age - RR ↑ |
| Dennis et al., Stroke, 2002^6^ | PC | United Kingdom | 2696 (5.6% hemorrhagic stroke) 129935 (% of hemorrhagic strokes n.a.) | 2  10 | MR - 1.41 (hip fractures within 2 years) [95%CI 0.9-2.1] MR - 1.68 (hip fractures within 10 years) [95% CI 1.6-1.7] Incidence of 22 per 1000 person-years [CI n.a.] | n.a. | no | older age ↑, female sex ↑, cognitive impairment ↑, pre stroke mRS >2 ↑ |
| Brown et al., Cerebrovasc Dis., 2008^7^ | RC | USA | 642 (5.6% hemorrhagic stroke) | 1 | 3.1% (all fractures) [CI n.a.] | n.a. | yes | none |
| Sennerby et al., JAMA, 2009^8^ | RC | Sweden | 2962 (% of hemorrhagic strokes n.a.) | 25 | HR 5.09 (hip fractures) [95%CI 4.2-6.2] HR 4.95 in ischemic strokes (hip fractures) [95%CI 4.1-6.0] | n.a. | no | female sex ↑, arterial hypertension ↑ |
| Pouwels et al., Stroke, 2009^9^ | RC | Netherlands | 6763 (15,6% hemorrhagic stroke) | 8 | OR 1.96 (hip fractures) [95%CI 1.7–2.3] | n.a. | no | female sex ↑, younger than 71 years ↑, short time to sustained stroke ↑ |
| Vestergaard et al., Calcif Tissue Int., 2009^10^ | RC | Denmark | 124655 (% of hemorrhagic strokes n.a.) | n.a. | OR 1.24 (any fractures) within 3 years [95%CI 1.2–1.3] OR 1.84 (hip fractures) within 3 years [95%CI 1.6–2.1] | n.a. | no | n.a. |
| Wu et al., Arch Phys Med Rehabil, 2011^11^ | PC | Taiwan | 1951 (ischemic stroke only) | 10 | HR 2.33 [95%CI 1.6–3.3] in women and HR 1.73 [95%CI 1.1–2.7] in men (hip fractures) | n.a. | no | female sex ↑ |
| Andersson et al., Stroke Res Treat, 2013^12^ | PC | Sweden | 377 (% of hemorrhagic strokes n.a.) | 2 | n.a. | n.a. | no | cognitive impairment ↑ |
| Lin et al., Acta Neurol Scand., 2015^13^ | RC | Taiwan | 18413 (14.2% hemorrhagic stroke) | 12 | 1.6-fold risk (hip fractures) [CI n.a.] | n.a. | no | older age ↑, female sex ↑, use of antidepressants ↑ |
| Benzinger et al., Osteoporos Int., 2015^14^ | RC | Germany | 78461 (% of hemorrhagic strokes n.a.) | 5 | HR 1.72 [95%CI 1.6-1.8] in men and HR 1.38 [95%CI 1.3-1.4] in women (all osteoporotic fractures) | n.a. | no | no severe functional impairment (RR) ↑ |
| Kapral et al., Neurology, 2017^15^ | RC | Canada | 23751 (14% hemorrhagic stroke) | 2 | HR 1.47 (any low-trauma fracture) [95%CI 1.4–1.6] HR 1.76 (femur fracture) [95%CI 1.6–2.0] | n.a. | yes^a^ | older age ↑, female sex ↑, higher stroke severity ↑, prior stroke ↑, atrial fibrillation ↑, rheumatoid arthritis ↑, hyperparathyroidism ↑, prior diagnosis of osteoporosis ↑, prior falls ↑, prior fractures ↑ |
| Foster et al., Front Neurol., 2018^16^ | PC | United Kingdom | 7267 (10.2% hemorrhagic stroke) | 10 | n.a. | n.a. | yes | older age ↑, female sex ↑, history of falls ↑, previous stroke/TIA ↑, prestroke mRS score 3–5 ↓ |
| Lee KB et al., J Korean Med Sci, 2019^17^ | RC | Korea | 11522 (ischemic stroke only) | 4 | 4.43% [95%CI 4.1–4.8] at 1 year, 13.00% [95%CI 12.4–13.6] at 4 years | n.a. | no | older age ↑, female sex ↑, osteoporosis ↑, previous fracture ↑ |
| Tanislav et al., Osteoporos Int., 2020^18^ | RC | Germany | 36795 (ischemic stroke only) | 5 | HR 1.26 - stroke (all fractures) [95%CI 1.2–1.4] HR 1.14 - TIA (all fractures) [95%CI 1.0–1.3] | n.a. | yes | older age ↑, female sex ↑, dementia ↑, non-opioid analgesic therapy ↑ |
| Kristensen et al., Acta Neurol Scand., 2020^19^ | RC | Denmark | 116519 (22.4% hemorrhagic stroke) | 14 | Incidence of 41.07 per 1000 person‐years (all fractures) [95%CI 40.4-41.7] | n.a. | no | Mild, moderate or severe stroke severity ↑, living alone ↑ |
| Dalli et al., Stroke, 2023^20^ | RC | Australia | 13594 (12.1% hemorrhagic stroke) | 1  before and after ischemic event | Incidence of 74 per 1000 person-years [95%CI 69–79] one year after stroke  in total 63% [95%CI 47%–80%] higher fracture rate compared to the year before the ischemic event | stroke: 47 per 1000 person-years (all fractures)  TIA: 41 per 1000 person-years | yes | older age ↑, female sex ↑, higher Charlson Comorbidity Index ↑, Stroke Unit care ↓ |

^a^ TIA patients were included, but no HR of fracture risk for TIA patients vs. controls was published

Abbreviations: RC, retrospective cohort; PC, prospective cohort; CI, confidence interval; RR, relative risk; MR, morbidity ratio; mRS, modified Rankin Scale; HR, hazard ratio.

**Supplementary references**

1. Ramnemark A, Nyberg L, Borssén B, et al. Fractures after stroke. *Osteoporos Int*. 1998;8(1):92-95.
2. Kanis J, Johnell O, Gullberg B, et al. Risk factors for hip fracture in men from southern Europe: the MEDOS study. Mediterranean Osteoporosis Study. *Osteoporos Int*. 1999;9(1):45-54.
3. Ramnemark A, Nilsson M, Borssén B, et al. Stroke, a major and increasing risk factor for femoral neck fracture. *Stroke*. 2000;31(7):1572-1577.
4. Melton LJ III, Brown RD Jr, Achenbach SJ, et al. Long-Term fracture risk following ischemic stroke: a population-based study*. Osteoporos Int*. 2001;12(11):980-986.
5. Kanis J, Oden A, Johnell O. Acute and long-term increase in fracture risk after hospitalization for stroke. *Stroke*. 2001;32(3):702-706.
6. Dennis MS, Lo KM, McDowall M, et al. Fractures after stroke: frequency, types, and associations. *Stroke*. 2002;33(3):728-734.
7. Brown DL, Morgenstern LB, Majersik JJ, et al. Risk of fractures after stroke. *Cerebrovasc Dis*. 2008;25(1-2):95-99.
8. Sennerby U, Melhus H, Gedeborg R, et al. Cardiovascular diseases and risk of hip fracture. *JAMA*. 2009;302(15):1666-1673.
9. Pouwels S, Lalmohamed A, Leufkens B, et al. Risk of hip/femur fracture after stroke: a population-based case-control study. *Stroke*. 2009;40(10):3281-3285.
10. Vestergaard P, Rejnmark L, Mosekilde L. Hypertension is a risk factor for fractures. *Calcif Tissue Int*. 2009;84(2):103-111.
11. Wu CH, Liou TH, Hsiao PL, et al. Contribution of ischemic stroke to hip fracture risk and the influence of gender difference. *Arch Phys Med Rehabil*. 2011;92(12):1987-1991.
12. Andersson AG, Seiger A, Appelros P. Hip fractures in persons with stroke. *Stroke Res Treat*. 2013;2013:954279.
13. Lin HL, Lin HC, Tseng YF, et al. Hip fracture after first-ever stroke: a population-based study. *Acta Neurol Scand*. 2015;131(3):158-163.
14. Benzinger P, Rapp K, König HH, et al. Risk of osteoporotic fractures following stroke in older persons. *Osteoporos Int*. 2015;26(4):1341-1349.
15. Kapral MK, Fang J, Alibhai SM, et al. Risk of fractures after stroke: Results from the Ontario Stroke Registry. *Neurology*. 2017;88(1):57-64.
16. Foster EJ, Barlas RS, Bettencourt-Silva JH, et al. Long-Term Factors Associated With Falls and Fractures Poststroke. *Front Neurol*. 2018;9:210.
17. Lee KB, Lee JG, Kim BJ, et al. The Epidemiology of Fracture in Patients with Acute Ischemic Stroke in Korea. *J Korean Med Sci*. 2019;34(22):e164.
18. Tanislav C, Kostev K. Factors associated with fracture after stroke and TIA: a long-term follow-up*. Osteoporos Int*. 2020;31(12):2395-2402.
19. Kristensen J, Birn I, Mechlenburg I. Fractures after stroke-A Danish register-based study of 106 001 patients. *Acta Neurol Scand*. 2020;141(1):47-55.
20. Dalli LL, Borschmann K, Cooke S, et al. Fracture Risk Increases After Stroke or Transient Ischemic Attack and Is Associated With Reduced Quality of Life. *Stroke*. 2023;54(10):2593-2601.
